# Data Resource Profile: Multimorbidity in Africa Digital Innovation, Visualisation and Application (MADIVA) research hub

**DOI:** 10.1093/ije/dyag124

**Published:** 2026-07-25

**Authors:** Daniel Ohene-Kwofie, Nkosinathi Masilela, Kerry Glover, Samuel Iddi, Daniel Maina, Molulaqhooa Linda Maoyi, Theophilous Mathema, Mwiza Gideon Singini, Scott Hazelhurst, Chodziwadziwa Whiteson Kabudula, MADIVA Research Hub and the DS-I Africa Consortium, Daniel Ohene-Kwofie, Nkosinathi Masilela, Kerry Glover, Samuel Iddi, Daniel Maina, Molulaqhooa Linda Maoyi, Theophilous Mathema, Mwiza Gideon Singini, Scott Hazelhurst, Chodziwadziwa Whiteson Kabudula

**Affiliations:** SAMRC/Wits Rural Public Health and Health Transitions Research Unit, School of Public Health, Faculty of Health Sciences, University of the Witwatersrand, Johannesburg, South Africa; School of Electrical and Information Engineering, University of the Witwatersrand, Johannesburg, South Africa; SAMRC/Wits Rural Public Health and Health Transitions Research Unit, School of Public Health, Faculty of Health Sciences, University of the Witwatersrand, Johannesburg, South Africa; Sydney Brenner Institute for Molecular Bioscience, Faculty of Health Sciences, University of the Witwatersrand, Johannesburg, South Africa; African Population and Health Research Center (APHRC), APHRC Campus, Nairobi, Kenya; Department of Statistics and Actuarial Science, University of Ghana, Legon, Accra, Ghana; African Population and Health Research Center (APHRC), APHRC Campus, Nairobi, Kenya; DSTI-SAMRC South African Population Research Infrastructure Network (SAPRIN), South African Medical Research Council, Durban, South Africa; Sydney Brenner Institute for Molecular Bioscience, Faculty of Health Sciences, University of the Witwatersrand, Johannesburg, South Africa; Non-Communicable Diseases Research Unit (NCDRU), South African Medical Research Council, Cape Town, South Africa; School of Electrical and Information Engineering, University of the Witwatersrand, Johannesburg, South Africa; Sydney Brenner Institute for Molecular Bioscience, Faculty of Health Sciences, University of the Witwatersrand, Johannesburg, South Africa; SAMRC/Wits Rural Public Health and Health Transitions Research Unit, School of Public Health, Faculty of Health Sciences, University of the Witwatersrand, Johannesburg, South Africa

**Keywords:** multimorbidity, sub-Saharan Africa, data harmonization, health and demographic surveillance system, data science, non-communicable diseases

Key Features

- The Multimorbidity in Africa Digital Innovation, Visualisation, and Application (MADIVA) research hub was established to facilitate the study of multimorbidity and associated risk factors, disease clustering, and stratification in African populations.
- The hub has harmonized and integrated data from population-based health and demographic surveillance system (HDSS) platforms from two sub-Saharan African settings representing both rural and urban populations.
- The platform consists of baseline population data collected from 1992 to 2024 in Agincourt, South Africa and from 2002 to 2019 in Nairobi, Kenya, with annual follow-ups and 10 nested health-related cohorts.
- The HDSS platforms contribute, overall, a total of >4 million person-years of observation from 574 598 individuals (54.8% females, 45.2% males). The harmonized nested cohorts contribute 130 550 observations on non-communicable diseases from 67 478 individuals (58.7% females, 41.3% males; mean age 44.5 years; 19.34 SD).
- The main categories of the data include socio-demographic characteristics, household and individual socio-economic status, and self-reported and objectively measured histories of chronic diseases and their treatments, as well as lifestyle behaviours. In addition, genotyping data are available for ∼6000 individuals.
- Data from the MADIVA hub provide a valuable resource for the longitudinal analyses of multimorbidity patterns, exploration of disease clustering, and the development of context-specific public health interventions.
- Interested researchers can contact the hub at info@madiva.africa or visit https://www.madiva.africa.

## Data resource basics

The burden of non-communicable diseases (NCDs) is rapidly increasing across Africa, driven by demographic and lifestyle transitions [1]. Concomitantly, levels of multimorbidity—commonly defined as the coexistence of two or more chronic conditions in an individual—are also rising [2, 3]. Multimorbidity is associated with increased premature mortality [4], lowered quality of life [5], diminished mental health [6, 7], increased risk of polypharmacy [8], and intensified health services utilization and associated costs, particularly in resource-poor settings [9]. Despite its significant impact, multimorbidity remains relatively under-researched compared with individual disease conditions [10].

Despite the complexities associated with multimorbidity and its rising burden in sub-Saharan Africa, most of the studies conducted have been in silos [11], making comparison of the findings difficult across the region. The systematic harmonization and integration of multiple datasets for multimorbidity may increase sample sizes and the comparability of study findings [12–14]. Additionally, data collected from longitudinal studies as well as Health and Socio-demographic Surveillance sites are a good source for understanding the dynamics of diseases in a population [15]. While individual datasets from various sources often capture similar constructs by using different measures, data harmonization enables comparability across these heterogeneous sources and facilitates their integration in a consistent and coherent manner. This facilitates cross-study comparisons to monitor, measure, and verify evolving population-level trends. In this data resource profile, we provide an overview of a novel data resource for the study of multimorbidity and associated risk factors in Africa produced by the Multimorbidity in Africa Digital Innovation, Visualisation and Application (MADIVA) research hub.

MADIVA is a research hub of the Data Science for Health Discovery and Innovation in Africa (DS-I Africa) Initiative. The goal of DS-I Africa is to leverage data science technologies to transform biomedical and behavioural research, including developing solutions that lead to improved health for individuals and populations in Africa. As part of MADIVA’s objectives, the hub is utilizing secondary data from two health and demographic surveillance system (HDSS) sites—rural Bushbuckridge (Agincourt), South Africa, and two urban informal settlements (Korogocho and Viwandani) of Nairobi, Kenya collected by the African Population and Health Research Center (APHRC)—to produce a harmonized data resource for the study of multimorbidity in Africa. Both sites have rich datasets from longitudinal and cohort studies gathered by the HDSS as well as nested research studies spanning several years. These existing data resources include demographic data, clinical health records, and genomic data, which are valuable for the study of multimorbidity. The aim of this paper is to discuss the data resource, with a particular emphasis on the analytical dataset created from multiple secondary data sources. It provides a comprehensive foundation for examining different health conditions, multimorbidity, and the associated health effects, including comprehensive studies of illnesses trends, risks factors, and long-term consequences across diverse populations.

## Data collected

The data included in the MADIVA data resource come from two primary sites. The first is the Agincourt HDSS (hereafter Agincourt), which is in the rural north-east of South Africa, close to the border with Mozambique. Agincourt was started in 1992 and currently includes an active population of ∼117 000 individuals from ∼22 000 households in 31 villages [16]. The second is the Nairobi Urban Health and Demographic Surveillance System (NUHDSS), which was established in 2002 in two slum communities in Nairobi (Korogocho and Viwandani). The NUHDSS platform investigates the long-term consequences of urban slum residence on health and socio-economic outcomes and, as of 2019, consisted of ∼88 974 active individuals from ∼33 462 households [17].

The current MADIVA database comprises representative, population-based, individual-level longitudinal and health surveys, including biomarkers, conducted in 2010 or later (for the Agincourt nested studies) and 2007 or later (for the NUHDSS nested studies). The research hub leveraged six studies (five longitudinal and one observational) from Agincourt and four longitudinal studies from the NUHDSS. Although all these nested studies were conducted independently at the respective sites at different time points, they all include biomarker information as well as self-reported responses that provide valuable information for the study of multimorbidity. In addition to the studies nested in the HDSS sites, the harmonization included socio-demographic and household information as well as individual event history from the respective HDSS platforms. Table 1 provides an overview of the various studies incorporated into the MADIVA data-harmonization process, detailing the data time points, volume, and types of data collected.

**Table 1:** Detailed description of the included projects providing data for the MADIVA harmonization and integration.

Project	Data collected	Study design	Countries	Data volume	Study period	Age (years) [mean (SD)]
Health and socio-demographic platform [16, 17, 23]	Demographic and social factors, health behaviours, medical history and exposures Supplementary Table 1 presents the detailed person-years and mortality rates by year for each of the sites	Longitudinal	South Africa and Kenya	South Africa: 35 912 households, 311 633 individuals, 31 villages Kenya: 84 903 households, 262 915 individuals	South Africa: annually since 1992 Kenya: 2 or 3 rounds per year from 2002 to 2019	All ages
Verbal autopsy (VA) [24]	The World health Organization (WHO)-based VA questionnaire providing data on symptoms of the illness or injury prior to death, including health-seeking behaviour for all deceased individuals to facilitate automated cause-of-death assignment Supplementary figure S1a and b provide broad causes of death for each HDSS site	Cross-sectional	South Africa and Kenya	South Africa: 21 270 deceased individuals Kenya: 5474 deceased individuals	South Africa: annually since 1992 Kenya: 2002–17	All ages: 48.7 (25.56); 32.2 (21.17)
Africa Wits-INDEPTH study of genomic and environmental factors for cardiometabolic risk (AWI-Gen 1,2) [25, 26]	Socio-demographic characteristics, lifestyle behaviour, medical history of chronic and infectious diseases, anthropometry, body fat distribution, blood pressure, and blood and urine biomarkers Genomic data	Longitudinal	South Africa and Kenya	South Africa: Wave 1 = 2486; Wave 2 = 1255 Kenya: Wave 1 = 2003; Wave 2 = 1189 4489 genotyped 30 whole-genome sequences	South Africa: 2014–15; 2019–21 Kenya: 2014–15; 2020–21	38+ years: 58.7 (11.03); 62.6 (10.54); 48.6 (5.85); 54.0 (5.74)
African Research on Kidney Disease (ARK) Study Phase 1 and 2 [27]	Socio-demographic, anthropometric, and clinical data; blood samples for creatinine, hepatitis B serology; spot urine for dipstick testing and urine albumin: point-of-care: HIV infection, diabetes, and hypercholesterolaemia Genomic data	Longitudinal	South Africa	South Africa: Wave 1 = 2759; Wave 2 = 1505 630 genotyped	South Africa: 2017–18 ; 2018–19	20–79 years: 37.2 (13.86); 41.2 (14.58)
Health and Aging in Africa: A Longitudinal Study of an INDEPTH Community in South Africa (HAALSI) 1, 2, 3 [28]	Demographic and social factors, health behaviours, anthropometric measurements, biomarkers, cognition, mental health, and self-reported health Genomic data	Longitudinal	South Africa	South Africa: Wave 1 = 5059; Wave 2 = 4176; Wave 3 = 3707 4000+ genotyped	South Africa: 2014–15; 2018–19; 2021–22	40+ years: 62.3 (13.00); 65.1 (12.57); 66.6 (12.00)
HIV/cardiometabolic risk factor survey—HIV NCD [29]	Sexual behaviour and chronic-disease risk factors; anthropometric measurements; and biomarkers for diabetes, cholesterol, and dried blood spots (DBS) for HIV	Cross-sectional	South Africa	4704 individuals	2010–11	15+ years: 42.0 (19.23)
Nkateko Trial—Hypertension Surveillance 1 and 2 [30]	Clinic-based chronic-disease data: HIV, hypertension, diabetes; physical measurements (blood pressure)	Longitudinal	South Africa	Wave 1 = 3772; Wave 2 = 3015	2013–14 ; 2014–15	17+ years: 56.2 (19.26); 52.3 (19.18)
Individual Health 1 and 2	Nested within the HDSS platform, collected anthropometric measurement (BMI), HIV status (rapid test + DBS), self-reported general health (HIV, diabetes, hypertension, TB)	Longitudinal	South Africa	Wave 1 = 36 809; Wave 2 = 37 278	2021–22; 2022–23	18+ years: 38.1 (18.82); 39.6 (19.65)
Assessing the links between socio-economic status, perceived personal risk, and risk factors for cardiovascular and related NCDs in a population of slum dwellers in Nairobi, Kenya [31]	Assessed the connections between socio-economic status (SES), perceived personal risk, and risk factors for cardiovascular and other NCDs	Longitudinal	Kenya	Alcohol = 106; main = 5396	2007–09; 2008–09	18+ years: 38.8 (15.80); 43.2 (14.89)
A community-based intervention for primary prevention of cardiovascular diseases in the slums of Nairobi: the SCALE-UP (SU) study protocol for a prospective quasi-experimental community-based trial (SU Population Baseline & Endline, SU follow-up clinic) [32]	Demographic and socio-economic data; anthropometric and clinical measurements including CVD behavioural risk factors, physical measurements (weight, height, waist and hip circumference, blood pressure), glucose	Longitudinal	Kenya	First clinic = 721; follow-up clinic = 3313; clinic baseline = 604; clinic end line = 509; population baseline = 5649; population end line=4466	2011–14; 2012–14 ; 2012–14; 2013–14; 2012–13; 2013–14	35+ years: 51.3 (17.89); 54.0 (15.29); 51.3 (17.38); 52.1 (16.77); 45.5 (12.78); 46.8 (9.35)
Diabetic (baseline and end line) [33]	Socio-demographic and clinical characteristics of study participants including health behaviour—diet (salt intake) and exercise, healthcare-seeking, as well as blood pressure, anthropometric measurements—BMI, waist, hip; fasting blood glucose	Longitudinal	Kenya	Baseline = 85; end line =47	2009–10; 2010–11	18+ years: 53.9 (15.58); 54.8 (12.89)

BMI, body mass index; CVD, cardiovascular disease; TB, tuberculosis; DBS, dried blood spots; WHO, World Health Organization.

Datasets from the indicated studies (see Table 1) and the HDSS were collated into a relational database and then harmonized by using Structured Query Language. The harmonization process was guided by a stepwise and iterative approach, involving data-specification definitions and approval. The data-specification document highlights the expected variables, variable naming conventions, and the value domains, such as 0—no, 1—yes, 333—don’t know, 444—refused, 888—not applicable, 999—missing, etc. The current version of the data-specification document is available at http://github.com/MADIVA-DSI/data. Based on the data specification, we documented the variables collected for each study, including identifying domains and core target variables for each domain relevant for the study of multimorbidity.

The dataset from each integrated study was then extracted based on the data specification. This complex process involved aligning variable definitions across the studies (i.e. identifying and reviewing which questions align across studies as per the data specification), checking data quality and ensuring consistency, especially for biomarker measures, documenting variable skip patterns, and confirming which responses were missing from respective studies.

After the extraction, the dataset went through a rigorous review, validation, and profiling process to identify any data-quality issues from the extraction process. Data quality was ensured through a structured, multilayered approach involving study-specific internal consistency checks, as well as review and audits of harmonized datasets. Initial cleaning addressed duplicates, implausible values, coding non-responses and missing values, and logical inconsistencies—particularly in core demographic and temporal variables such as age, sex, and event dates (e.g. birth, death, and diagnosis). The data-profiling stage provided descriptive statistics with graphical representations for each variable enabling seamless review such as range checks, frequency distributions, and cross-tabulations to detect anomalies and verify the integrity of transformed and integrated variables. To assess completeness, we also calculated variable-level missingness across sites and time points, and visualized patterns to identify potential biases. Where necessary, the original metadata, data dictionaries, and survey instruments were consulted to support variable interpretation, with direct input from local data managers to resolve context-specific discrepancies. These quality-control procedures were repeated iteratively during harmonization, enhancing the reliability and comparability of the integrated datasets.

**Figure 1:**
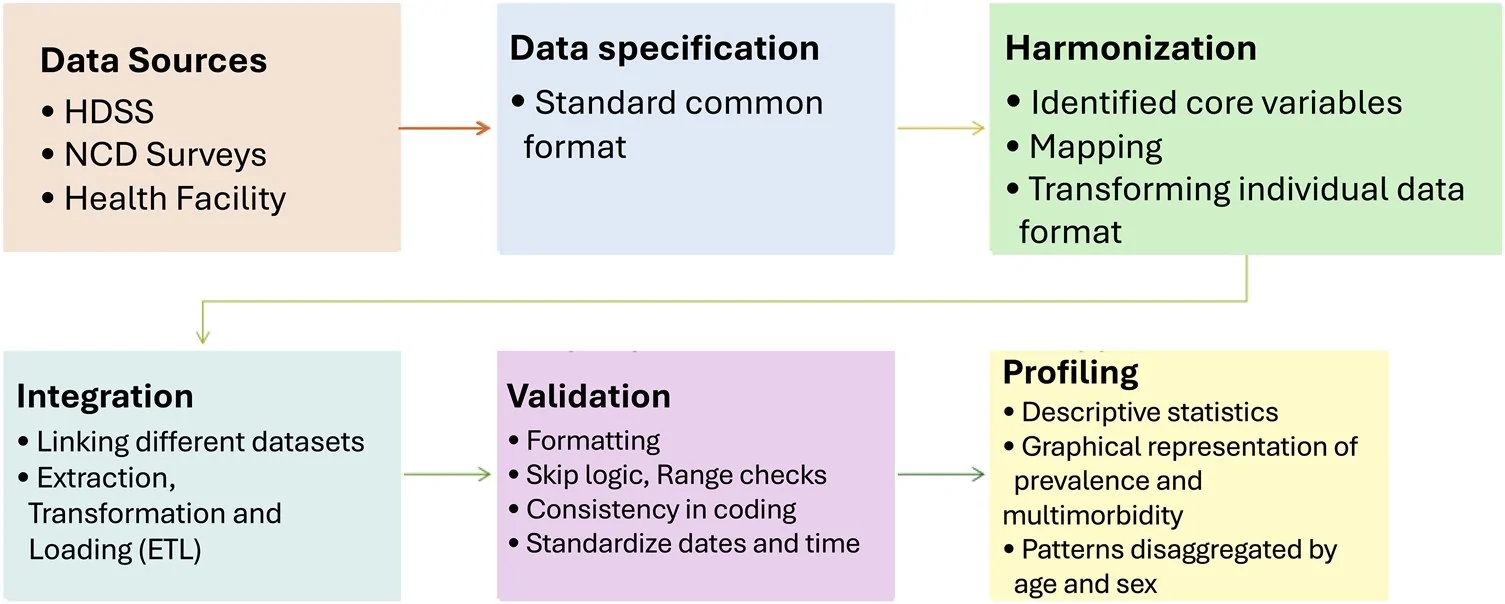
MADIVA data-harmonization and data-integration process. ETL, Extract, Transform, Load.

The finalized datasets in the relational database were imported into STATA, where the variables were carefully documented based on the data specifications. The resulting documented STATA dataset was used to generate comprehensive metadata documentation based on the Data Documentation Initiative (DDI) [18] standard and the international standard for data and metadata exchange using the DDI Metadata Editor (Nesstar Publisher [19]). The current version of the MADIVA DDI documentation is available at the repo http://github.com/MADIVA-DSI/data. The integration and harmonization processes are summarized in Fig. 1. A profile of the main categories of datasets generated from the integration and harmonization process is presented in Table 2.

**Table 2:** Profile of the data categories generated as part of the MADIVA database highlighting the data volume by sex for each HDSS site.

				Agincourt HDSS, South Africa			NUHDSS, Nairobi		
Dataset	Description	Variable count	Number of records (*N*)	Male (%)	Female (%)	*N* (%)	Male (%)	Female (%)	*N* (%)
Individuals	This dataset comprises demographic information on all unique individuals from the HDSS, including when they entered the surveillance area and when they moved out of the surveillance area	26	574 598 (∼4 million person-years)	139 428 (44.73)	172 243 (55.26)	311 677 (54.24)	120 520 (45.84)	142 395 (54.16)	262 921 (45.76)
Individual events	Provides the detailed event history observations of all individuals within the HDSS, including when and how they entered the surveillance (either through enumeration, birth or in-migration), as well as their current status within the HDSS (i.e. still current within the HDSS, moved out, or died)	7	164 5535	287 370 (44.55)	357 638 (55.45)	630 042 (38.28)	466 470 (45.94)	549 023 (54.06)	1 015 493 (61.71)
Individual socio-economic (SES) indicators	The dataset provides observations on the education, employment and marital status of individuals within the HDSS, captured across surveillance rounds	6	681 1985	1 179 931 (47.81)	1 287 988 (52.19)	2 464 207 (36.17)	2 043 496 (46.67)	2 334 860 (53.33)	4 347 778 (63.83)
Household memberships	The dataset consists of the membership observation records of everyone within the HDSS, linking individuals to their household and indicating their relationship with the head of the household for each episode	8	733 640	160 902 (44.94)	197 133 (55.06)	358 035 (48.80)	170 303 (45.34)	205 302 (54.66)	375 605 (51.20)
Households	Households describes the socio group for the individuals within the surveillance	4	120 815	N/A^1^	N/A	35 912 (29.72)	N/A	N/A	84 903 (70.28)
Household assets	This provides details about the asset socio-economic status (SES) of households within the surveillance. The household SES dataset includes 117 asset indicators	117	705 984	N/A	N/A	251 629 (35.64)	N/A	N/A	454 355 (64.36)
Verbal autopsy (VA)	The VA dataset includes data from the WHO standard questionnaire for all deaths occurring among the surveillance population, including InsilicoVA causes of death assignment	367	26 743	10 904 (51.26)	10 361 (48.71)	21 271 (79.54)	2329 (42.56)	3137 (57.33)	5472 (20.46)
NCD indicators	The NCD indicators dataset consists of observations from harmonized datasets from the 10 independent health-related longitudinal studies nested within the surveillance population. The data specification identified 396 variables across the different studies. Table 1 presents a detailed description of each of the studies integrated as part of this dataset	396	130 550	42 248 (39.64)	64 337 (60.36)	106 585 (81.64)	11 688 (48.77)	12 277 (51.23)	23 965 (18.36)

For the ‘Individuals’ and ‘Verbal autopsy (VA)’ datasets, ‘Number of records (*N*)’ refers to unique individuals, whereas it refers to unique household records for the ‘Households’ dataset. The ‘Number of records (*N*)’, however, refers to observations captured at each visit for the ‘Individual events’, ‘Individual socio-economic (SES) indicators’, ‘Household memberships’, ‘Household assets’, and ‘NCD indicators’ datasets.

N/A, not applicable; WHO, World Health Organization.

### The NCD indicators dataset

To facilitate the study of multimorbidity in Africa, the NCD indicators dataset leveraged data from existing health-related nested studies, which had their own research objectives but shared a common interest in NCDs, human immunodeficiency virus (HIV), and biomarkers, and their relationship with socio-demographic factors of the surveillance populations covered. The dataset was collated by the Data Management and Analytics Core of the MADIVA research hub, to meet the need for valid, reliable, and well-curated and well-documented data to help to understand, characterize the burden, and address the complexities associated with multimorbidity in Africa. Currently, 67 478 (∼12%) of the combined HDSS unique individuals have at least one observation from these NCD and health surveys, which were nested within the respective HDSS sites. The NCD and health surveys contribute a total of 130 550 observations, mainly among individuals aged ≥20 years, with close to 59% being observations from females. The participants included residents of NUHDSS (with 11 864 unique individuals who contributed 23 965 observations) and Agincourt (with 55 614 unique individuals who contributed 106 585 observations).

The variables harmonized and integrated from these surveys are broadly categorized into the following domains:

- socio-demographic: sex, age, marital status, educational level, employment status;
- health examinations and measurements such as blood pressure, pulse, height and weight, body mass index (BMI), waist and hip circumference;
- physical-activity status: activities of daily living, vigorous, moderate exercise, sedentary activity, pain;
- self-reported health conditions: diagnosis and treatment (including traditional treatment) of HIV, hypertension, diabetes, tuberculosis (TB), stroke, angina, high cholesterol, kidney disease, asthma, cancers, thyroid disease;
- healthcare utilization: number of health-facility/clinic visits;
- behavioural risk factors and health behaviours: tobacco use, alcohol consumption, diet (fruit, vegetables, soft drinks);
- sexual behaviour: condom use, number and types of sexual partners;
- point-of-care and laboratory results: blood assays: total cholesterol, high-density lipoprotein, low density lipoprotein, triglycerides, glucose, haemoglobin, HIV (viral load, antiretroviral therapy).

More information describing the data categories and variables can be found on page 4 of the Supplementary Material, available as Supplementary data at *IJE* online.

## Data resource use

The harmonized MADIVA dataset has only recently been assembled, making it a new and promising resource for studying NCDs and multimorbidity within HDSS populations across both rural and urban settings in sub-Saharan Africa. Although there have been a few publications based on the harmonized dataset [20–22], several analyses are currently underway. Initial work focuses on examining the prevalence and patterns of multimorbidity across the surveillance populations and assessing the trajectories of major chronic conditions and cardiometabolic risk factors contributing to multimorbidity, including HIV, hypertension, diabetes, stroke, and obesity.

Ongoing and planned analyses also include the application of machine-learning approaches, including auto-stratification techniques, to identify subpopulations with distinct risk profiles and disease trajectories. These methods are being used to explore the clustering of risk factors and conditions, improve the prediction of NCD outcomes, and identify groups at higher risk of adverse health outcomes. Further planned work includes analyses of mortality patterns, as well as disease-specific investigations aimed at improving the prediction and characterization of NCD risk and multimorbidity.

Beyond these, the harmonized dataset provides a valuable platform for addressing broader questions in epidemiology, population health, and health systems research. By integrating data from multiple longitudinal studies nested within HDSS platforms, the resource enables comparative and longitudinal analyses that are difficult to undertake by using individual studies alone.

### Chronic conditions and multimorbidity

**Figure 2:**
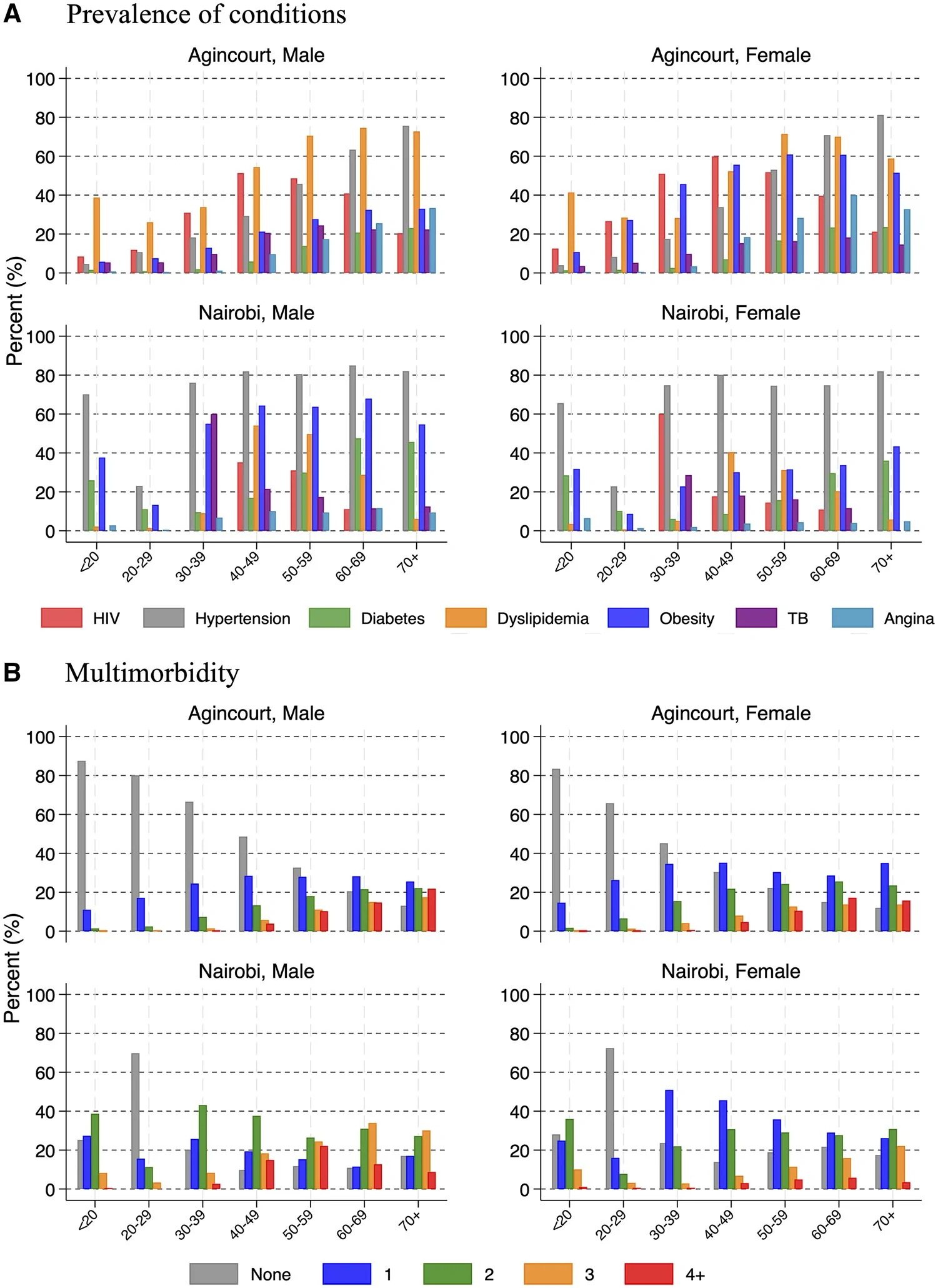
Age- and sex-specific distribution of chronic conditions and multimorbidity across sites. (a) Prevalence of selected chronic conditions (HIV, hypertension, diabetes, dyslipidaemia, obesity, tuberculosis, and angina) by age group and sex in Agincourt and Nairobi Urban surveillance sites. (b) Distribution of the number of chronic conditions per individual by age group and sex in Agincourt and Nairobi Urban surveillance sites.

Among the 67 478 unique individuals who contributed to the 130 550 NCD indicator dataset observations, 53% (35 876) suffered from at least one of the following conditions: HIV (19.7%), hypertension (35.6%), diabetes (6.3%), heart disease/failure (3.0%), stroke (6.0%), dyslipidaemia (43.9%), TB (3.4%), any cancers (4.8%), angina (12.8%), kidney disease/failure (7.3%), liver disease (1.2%), asthma (11.5%), thyroid disease (2.4%), and obesity (34.1%). Figure 2a highlights the prevalence of the top seven conditions and their differences by sex, age, and the respective HDSS sites. The figure generally shows increasing prevalence with age, particularly from the Agincourt HDSS. Furthermore, as many as 27% have two or more conditions, with observed differences across sex and age groups. Figure 2b summarizes the differences by age and sex, and Supplementary Table 3 provides details on the number of conditions across various socio-demographic characteristics. Supplementary Figure 3 presents the counts for the top 25 disease combinations.

## Strengths and weaknesses

### Strengths

The harmonized MADIVA database represents one of the first coordinated efforts to integrate longitudinal health and socio-demographic data across multiple population-based studies in South Africa and Kenya. The inclusion of both rural and peri-urban populations allows contextual comparisons and insights into geographic disparities in multimorbidity, including variation across demographic and socio-economic groups within the two countries. The standardization of core variables across distinct and independent studies enhances the analytic consistency of the dataset and supports cross-site, cross-country, and longitudinal analyses. The dataset is well suited for tracking multimorbidity patterns over time, investigating disease clustering, and informing context-specific public health interventions. An additional strength of the MADIVA database is the inclusion of biomarker and biological measurement data across contributing studies. These include blood pressure, blood glucose, lipid profiles, and anthropometric indicators—core variables for defining cardiovascular and metabolic risk factors central to the study of multimorbidity. This makes the MADIVA database a valuable population-based resource for epidemiological analyses, including disease clustering and risk stratification. Moreover, the integration of the HDSS dataset adds depth to the database by incorporating longitudinal socio-economic indicators at both individual and household levels. This facilitates nuanced exploration of the interplay between socio-economic status and the burden, distribution, and care pathways associated with multimorbidity in diverse African contexts. In addition, the dataset can reliably be used for mortality analysis across both sites (2002–15 for Nairobi Urban HDSS and 1992 to date for the Agincourt HDSS). Furthermore, work is ongoing to map the database to the Observational Medical Outcomes Partnership Common Data Model.

### Weaknesses

The limitations of the MADIVA database primarily stem from the inherent challenges of harmonizing heterogeneous measures across independent studies conducted in distinct geographic and cultural settings. Additionally, variation exists in the collection of certain biomarkers—such as those used to define diabetes—due to differences in the fasting-status protocols, measurement techniques (e.g. point-of-care versus laboratory-based testing), and the instruments used across the different studies and sites. These methodological differences may influence cross-site comparability. To address this, we have systematically documented all survey-specific measurement nuances in the data-specification document to support the transparent interpretation of findings.

Self-reported measures can be influenced by recall and reporting biases, which are affected by respondents’ education or health literacy. Although this may have led to the underestimation of disease prevalence or care-seeking behaviours, particularly in resource-limited settings, most studies included follow-up questions on treatment and care, to enhance accuracy.

## Data resource access

Access to the MADIVA data is available with approval from the Research Ethics Committee at the University of the Witwatersrand, South Africa. Researchers wishing to access this data resource may contact the principal investigator, Professor Scott Hazelhurst (Scott.Hazelhurst@wits.ac.za). The data dictionary for the integrated, harmonized, de-identified participant-level MADIVA database, including the database schema and data-specification document, is available at http://github.com/MADIVA-DSI/data. Additional information about MADIVA, including details on collaboration and data access, is also provided at https://www.madiva.africa/.

## Ethics approval

The University of the Witwatersrand Human Research Ethics Committee (Medical) (HREC M210825) and the Kenyan AMREF Research and Ethics and Scientific Review Committee (AMREF-ESRC P1206/2022) have approved MADIVA’s projects.

## Supplementary Material

dyag124_Supplementary_Data

## Data Availability

See ‘Data resource access’, above.
