# Supplementary material for: Data Resource Profile: Multimorbidity in Africa Digital Innovation, Visualisation and Application (MADIVA) research hub
Source: Int J Epidemiol. 2026 Jul 25;55(4):dyag124. doi: 10.1093/ije/dyag124 (PMC13401479; doi:10.1093/ije/dyag124)
Supplement: dyag124_Supplementary_Data [file dyag124_supplementary_data.zip › ije-2025-11-2161-File005.docx]

Supplementary Material

Table of Contents

[Mortality profile of MADIVA cohort 2](#_Toc233414458)

[Agincourt: Broad causes of death 4](#_Toc233414459)

[Nairobi: Broad causes of death 5](#_Toc233414460)

[The NCD Indicators dataset domains 6](#_Toc233414461)

[Introduction 6](#_Toc233414462)

[HDSS and Socio-demographic and economic domains 6](#_Toc233414463)

[Physical activity status domain 6](#_Toc233414464)

[Behavioural risk factors and health behaviours 6](#_Toc233414465)

[Health examinations and point of care domain 7](#_Toc233414466)

[Self-reported health conditions domain 8](#_Toc233414467)

[Statistical analyses & Multimorbidity 10](#_Toc233414468)

## Mortality profile of MADIVA cohort

Table S2: Mortality rates and person-time contribution by year from the Agincourt HDSS and Nairobi Urban HDSS^[[1]](#footnote-1)^.

|  | **Agincourt HDSS** | | | **Nairobi UHDSS** | | |
| --- | --- | --- | --- | --- | --- | --- |
| **Year** | Number Deaths | Person-Years | Deaths  Per 1000 PY | Number Deaths | Person-Years | Deaths  Per 1000 PY |
| 1993 | 316 | 65529.4 | 4.8 (4.3-5.4) | - | - | - |
| 1994 | 312 | 66446.3 | 4.7 (4.2-5.2) | - | - | - |
| 1995 | 353 | 67075.8 | 5.3 (4.7-5.8) | - | - | - |
| 1996 | 333 | 67588.7 | 4.9 (4.4-5.5) | - | - | - |
| 1997 | 314 | 68247.8 | 4.6 (4.1-5.1) | - | - | - |
| 1998 | 432 | 68916.2 | 6.3 (5.7-6.9) | - | - | - |
| 1999 | 443 | 70160.6 | 6.3 (5.8-6.9) | - | - | - |
| 2000 | 487 | 70931.6 | 6.9 (6.3-7.5) | - | - | - |
| 2001 | 582 | 71368.7 | 8.2 (7.5-8.8) | - | - | - |
| 2002 | 701 | 71460.6 | 9.8 (9.1-10.6) | - | - | - |
| 2003 | 803 | 71658.0 | 11.2 (10.5-12.0) | 535 | 57349.1 | 9.3 (8.6-10.2) |
| 2004 | 777 | 71995.0 | 10.8 (10.1-11.6) | 457 | 58661.4 | 7.8 (7.1-8.5) |
| 2005 | 847 | 72741.8 | 11.6 (10.9-12.5) | 453 | 61395.7 | 7.4 (6.7-8.1) |
| 2006 | 886 | 73756.7 | 12.0 (11.2-12.8) | 451 | 63607.4 | 7.1 (6.5-7.8) |
| 2007 | 906 | 75388.9 | 12.0 (11.3-12.8) | 438 | 65301.1 | 6.7 (6.1-7.4) |
| 2008 | 1030 | 87454.0 | 11.8 (11.1-12.5) | 423 | 66138.5 | 6.4 (5.8-7.0) |
| 2009 | 920 | 89251.5 | 10.3 (9.7-11.0) | 464 | 71112.5 | 6.5 (6.0-7.1) |
| 2010 | 827 | 90811.8 | 9.1 (8.5-9.7) | 494 | 73305.8 | 6.7 (6.2-7.4) |
| 2011 | 793 | 92406.3 | 8.6 (8.0-9.2) | 537 | 75052.0 | 7.2 (6.6-7.8) |
| 2012 | 757 | 93932.0 | 8.1 (7.5-8.7) | 427 | 73853.0 | 5.8 (5.3-6.4) |
| 2013 | 845 | 111113.7 | 7.6 (7.1-8.1) | 384 | 71214.8 | 5.4 (4.9-6.0) |
| 2014 | 857 | 116023.7 | 7.4 (6.9-7.9) | 370 | 69593.8 | 5.3 (4.8-5.9) |
| 2015 | 883 | 117414.5 | 7.5 (7.0-8.0) | 320 | 63223.2 | 5.1 (4.5-5.6) |
| 2016 | 880 | 118090.5 | 7.5 (7.0-8.0) | 163 | 56303.0 | 2.9 (2.5-3.4) |
| 2017 | 767 | 118380.5 | 6.5 (6.0-7.0) | 150 | 53491.3 | 2.8 (2.4-3.3) |
| 2018 | 792 | 118409.6 | 6.7 (6.2-7.2) | 124 | 52350.7 | 2.4 (2.0-2.8) |
| 2019 | 735 | 118474.7 | 6.2 (5.8-6.7) | - | - | - |
| 2020 | 767 | 118969.9 | 6.4 (6.0-6.9) | - | - | - |
| 2021 | 1047 | 118783.2 | 8.8 (8.3-9.4) | - | - | - |
| 2022 | 750 | 117010.6 | 6.4 (6.0-6.9) | - | - | - |
| 2023 | 704 | 116571.3 | 6.0 (5.6-6.5) | - | - | - |
|  |  |  |  |  |  |  |

Agincourt: Broad causes of death

Figure S1 (a): Percentage of deaths by broad causes and surveillance period, 1993–2023, disaggregated by age group for the Agincourt HDSS.

### Nairobi: Broad causes of death

Figure S1 (b): Percentage of deaths by broad causes ^[[2]](#footnote-2)^and surveillance period, 2003–2018, disaggregated by age group for the Nairobi Urban HDSS.

## The NCD Indicators dataset domains

### Introduction

We provide detailed information about the various variable and indicator domains from the respective studies and cohorts that have been integrated. Figure S1 illustrates the number of health-related indicators available from each of the integrated studies.

### HDSS and Socio-demographic and economic domains

Supplementary Table S1 shows the various domains and the indicator variables, across the different datasets used for the harmonisation. Harmonisation of the de-identified socio-demographic and economic variables was straightforward across all studies, as well as the HDSS dataset. The harmonised education variable was created by converting the education level into the number of years of education, while employment status and marital status were seamlessly retrieved from the HDSS data for the various study participants.

### Physical activity status domain

The physical activity domain involved questions about whether the participant’s daily routine involves moderate to vigorous intense activity (either work, transport, and/or leisure related), including sporting, and cycling activities. The questions also involve the hours spent on sleeping.

### Behavioural risk factors and health behaviours

Behavioural risk factors and health behaviours such as tobacco consumption, alcohol use and diet were also harmonised across studies. The questions asked about “ever used tobacco (including cigarettes— manufactured or rolled, pipes, cigars, snuff or chewing tobacco)”, as well as “Do you currently smoke tobacco products daily?” to capture current smoking status.

Alcohol consumption data was also available and included questions on whether participants had ever consumed alcohol, the type, and quantity of alcohol, as well as the frequency of consumption.

Additionally, information on dietary consumption, including frequency, quantity of fruit, and vegetables was collected by some studies. The Diabetic baseline study provided additional questions regarding the consumption of soft drinks, salt, oil, meat, chips, and other dietary items that were not covered by other studies. These variables have been incorporated into the data specification to ensure comprehensive dietary information for the relevant datasets.

These datasets also included variables on sexual behaviours such as condom use, transactional sex, and the number and types of sexual partners. Participants also reported whether they had ever been tested for HIV and if they had engaged in unprotected sex with HIV positive partners. Additional details of variables for the respective domains are available in the Supplementary Table S1.

### Health examinations and point of care domain

The various datasets included a comprehensive set of measures such as weight, height, hip and waist circumferences, blood pressure; and point of care and dried blood spot. Blood pressure (systolic, diastolic) measurements were available for all nested studies. For each study, the average of the second and third measurements or the available measurements were taken to classify hypertensive status as mean systolic blood pressure ≥ 140 mmHg or diastolic blood pressure ≥ 90 mmHg or ever been/or currently on hypertension treatment. Height, hip, and waist circumference measurements were standardised to centimetres (cm), while weight to kilogram (kg), and pulse standardised to beats per minute (bpm). Body Mass Index (BMI) was computed using available height and weight measurements. Supplementary Table S1 highlights the indicators and the respective study sources, as well as the study domain.

Additionally, point-of- care total cholesterol, low-density lipoprotein (LDL) cholesterol, high-density lipoprotein (HDL) cholesterol and triglycerides (all in mmol/L) as well as haemoglobin were available from several studies.

### Self-reported health conditions domain

The variables in this domain contain information about ever been diagnosed, currently diagnosed, ever been on treatment or currently on treatment including, medical history of chronic and infectious diseases such as HIV, hypertension, diabetes, TB, liver disease, stroke, any cancers, asthma, thyroid disease, kidney disease/infection, heart disease/failure, angina. For each study, the questions asked include “Have you ever been diagnosed with/told by a doctor that you have […]?” or “Ever been treated for […] / Have you ever seen a traditional healer/herbalist for […]?” or “newly diagnosed with […] in the last 12 months? / Have you been told in the past 12 months for the first time?”. These self-reported conditions were available in some form across all studies. Further details about the actual questions across the different studies are available from MADIVA data specification document at http://github.com/MADIVA-DSI/data.

In addition to the self-reported conditions, we created derived variables to provide a broad definition of some conditions, particularly chronic conditions, based on objective measurements and laboratory tests results from the respective studies. For hypertension, in addition to self-reported status, systolic blood pressure  ≥ 140 mmHg or diastolic systolic blood pressure  ≥ 90 mmHg, are coded as hypertensive; for diabetes (elevated glucose - non-fasting  ≥  11.1mmol/L, or fasting >7mmol/L) ; for dyslipidemia (total cholesterol > 6.21 mmol/L or LDL > 4.1 mmol/L or HDL < 1.19 mmol/L or triglycerides > 1.7 mmol/L); obesity (BMI  ≥  30) ; as well as HIV test results. For the chronic conditions: HIV, hypertension, diabetes, stroke, cancers, heart disease, angina, kidney disease, TB, liver disease, asthma, thyroid disease, obesity, and dyslipidemia, we created a derived status based on self-report of ever been diagnosed, currently diagnosed, currently or ever been on treatment for the respective conditions. We proceeded to impute subsequent observations for each individual based on the results of the conditions identified at the initial time point. For example, if an individual had three observations from different data sources and the first source included an HIV question or a test indicating a positive result, while the subsequent sources did not include such questions or results, we imputed the remaining two observations to reflect the individual's status prospectively.

Figure S2 : The number of available health related variables or indicators from each of the studies from the respective HDSS sites harmonised and integrated as part of the MADIVA database.

## Statistical analyses & Multimorbidity

We present a general description of the baseline characteristics of the unique individuals from the integrated HDSS study population, and their differences by general socio-demographic characteristics such as age, sex, marital status, years of education, and employment status. We grouped age into 5-year age groups; marital status into: never married, separated or divorced, widowed and currently married). The years of education were grouped into: no formal education, incomplete primary (1-6 years), complete primary (7 years), incomplete secondary (8-11 years), complete secondary (12 years), and college/university (13+ years). The employment status, on the other hand, was a simple ‘yes’ or ‘no’ variable to indicate whether the individual was working or not. We also included variables to determine whether they were deceased (died-yes/no), as well as when they moved into and out of the surveillance area, grouped into 5-year intervals. We further explore the characteristics of individuals with at least one chronic condition, as well as multiple disease combinations, to understand the burden of multimorbidity from the integrated dataset.

Descriptive statistics were expressed as mean and standard error (SE) for continuous variables and as frequency for categorical variables. All statistical analyses were performed using Stata Statistical Software (STATA) version 18.5 (Stata Corporation, College Station, TX, USA, 2025). Table S3 shows multimorbidity by various socio-demographic characteristics of the MADIVA data.

Table S3: Multimorbidity and socio-demographic characteristics of the MADIVA data

|  | Number of conditions | | | | | |  |
| --- | --- | --- | --- | --- | --- | --- | --- |
|  | **None** | **1 Condition** | **2 Conditions** | **3 Conditions** | **4+ Conditions** | **Total** | ***p-value*** |
|  | *n = 31594 (%)* | *n = 17868 (%)* | *n = 10275 (%)* | *n = 4331 (%)* | *n = 3410 (%)* | *n = 67478 (%)* |  |
| **Sex** |  |  |  |  |  |  | *** |
| Male | 15346 (48.6) | 5704 (31.9) | 3551 (34.6) | 1780 (41.1) | 1484 (43.5) | 27865 (41.3) |  |
| Female | 16248 (51.4) | 12164 (68.1) | 6724 (65.4) | 2551 (58.9) | 1926 (56.5) | 39613 (58.7) |  |
| **Age Group** |  |  |  |  |  |  | *** |
| < 19 | 8045 (25.5) | 1279 (7.2) | 287 (2.8) | 42 (1.0) | 6 (0.2) | 9659 (14.3) |  |
| 20-24 | 6332 (20.0) | 1668 (9.3) | 314 (3.1) | 48 (1.1) | 6 (0.2) | 8368 (12.4) |  |
| 25-29 | 4654 (14.7) | 1620 (9.1) | 477 (4.6) | 104 (2.4) | 8 (0.2) | 6863 (10.2) |  |
| 30-34 | 3548 (11.2) | 1682 (9.4) | 653 (6.4) | 165 (3.8) | 15 (0.4) | 6063 (9.0) |  |
| 35-39 | 2370 (7.5) | 2385 (13.3) | 1377 (13.4) | 291 (6.7) | 70 (2.1) | 6493 (9.6) |  |
| 40-44 | 1803 (5.7) | 2044 (11.4) | 1441 (14.0) | 442 (10.2) | 277 (8.1) | 6007 (8.9) |  |
| 45-49 | 1371 (4.3) | 1716 (9.6) | 1231 (12.0) | 539 (12.4) | 399 (11.7) | 5256 (7.8) |  |
| 50-54 | 1128 (3.6) | 1415 (7.9) | 1091 (10.6) | 572 (13.2) | 420 (12.3) | 4626 (6.9) |  |
| 55-59 | 789 (2.5) | 999 (5.6) | 884 (8.6) | 525 (12.1) | 480 (14.1) | 3677 (5.4) |  |
| 60-64 | 535 (1.7) | 851 (4.8) | 749 (7.3) | 435 (10.0) | 459 (13.5) | 3029 (4.5) |  |
| 65-69 | 372 (1.2) | 657 (3.7) | 591 (5.8) | 380 (8.8) | 385 (11.3) | 2385 (3.5) |  |
| 70-74 | 218 (0.7) | 481 (2.7) | 409 (4.0) | 288 (6.6) | 353 (10.4) | 1749 (2.6) |  |
| 75+ | 429 (1.4) | 1071 (6.0) | 771 (7.5) | 500 (11.5) | 532 (15.6) | 3303 (4.9) |  |
| **Marital Status** |  |  |  |  |  |  | *** |
| Single/Never married | 22934 (72.6) | 7846 (43.9) | 2718 (26.5) | 812 (18.7) | 436 (12.8) | 34746 (51.5) |  |
| Cohabiting | 1936 (6.1) | 1416 (7.9) | 703 (6.8) | 266 (6.1) | 198 (5.8) | 4519 (6.7) |  |
| Currently married | 4560 (14.4) | 5672 (31.7) | 4402 (42.8) | 1911 (44.1) | 1432 (42.0) | 17977 (26.6) |  |
| Separated | 1208 (3.8) | 1103 (6.2) | 1056 (10.3) | 585 (13.5) | 464 (13.6) | 4416 (6.5) |  |
| Divorced | 307 (1.0) | 436 (2.4) | 331 (3.2) | 181 (4.2) | 209 (6.1) | 1464 (2.2) |  |
| Widowed | 536 (1.7) | 1246 (7.0) | 900 (8.8) | 537 (12.4) | 662 (19.4) | 3881 (5.8) |  |
| Not applicable | 7 (0.0) | 51 (0.3) | 87 (0.8) | 18 (0.4) | 4 (0.1) | 167 (0.2) |  |
| Unknown/Missing | 106 (0.3) | 98 (0.5) | 78 (0.7) | 21 (0.5) | 5 (0.1) | 308 (0.5) |  |
| **Years of Education** |  |  |  |  |  |  | *** |
| No formal education | 1324 (4.2) | 2387 (13.4) | 1920 (18.7) | 1187 (27.4) | 1257 (36.9) | 8075 (12.0) |  |
| Incomplete primary (1-6 years) | 1725 (5.5) | 1995 (11.2) | 1558 (15.2) | 866 (20.0) | 810 (23.8) | 6954 (10.3) |  |
| Complete primary (7 years) | 1944 (6.2) | 2186 (12.2) | 1786 (17.4) | 671 (15.5) | 487 (14.3) | 7074 (10.5) |  |
| Incomplete secondary (8-11 years) | 10752 (34.0) | 4369 (24.5) | 2030 (19.8) | 786 (18.1) | 457 (13.4) | 18394 (27.3) |  |
| Complete secondary(12 years) | 12505 (39.6) | 5758 (32.2) | 2513 (24.5) | 663 (15.3) | 287 (8.4) | 21726 (32.2) |  |
| College/University (13+ years) | 3049 (9.7) | 963 (5.4) | 318 (3.1) | 119 (2.7) | 98 (2.9) | 4547 (6.7) |  |
| Unknown/Missing | 295 (0.9) | 210 (1.2) | 150 (1.4) | 39 (0.9) | 14 (0.4) | 708 (1.1) |  |
| **Year into DSA** |  |  |  |  |  |  | *** |
| 1992-1996 | 3887 (12.3) | 3503 (19.6) | 2280 (22.2) | 1422 (32.8) | 1608 (47.2) | 12700 (18.8) |  |
| 1997-2001 | 2864 (9.1) | 1403 (7.9) | 662 (6.4) | 334 (7.7) | 322 (9.4) | 5585 (8.3) |  |
| 2002-2006 | 6889 (21.8) | 4217 (23.6) | 3186 (31.0) | 1343 (31.0) | 868 (25.5) | 16503 (24.5) |  |
| 2007-2011 | 6495 (20.6) | 3521 (19.7) | 1970 (19.2) | 702 (16.2) | 514 (15.1) | 13202 (19.6) |  |
| 2012-2016 | 8664 (27.4) | 4101 (23.0) | 1841 (17.9) | 471 (10.9) | 95 (2.8) | 15172 (22.5) |  |
| 2017-2021 | 2758 (8.7) | 1115 (6.2) | 336 (3.3) | 59 (1.4) | 3 (0.1) | 4271 (6.3) |  |
| 2022-2024 | 37 (0.1) | 8 (0.0) | 0 (0.0) | 0 (0.0) | 0 (0.0) | 45 (0.1) |  |
| **Year out of DSA** |  |  |  |  |  |  | *** |
| 1992-1996 | 4 (0.0) | 6 (0.0) | 3 (0.0) | 0 (0.0) | 2 (0.1) | 15 (0.0) |  |
| 1997-2001 | 7 (0.0) | 6 (0.0) | 4 (0.0) | 3 (0.1) | 1 (0.0) | 21 (0.0) |  |
| 2002-2006 | 20 (0.1) | 23 (0.1) | 18 (0.2) | 3 (0.1) | 2 (0.1) | 66 (0.1) |  |
| 2007-2011 | 1174 (3.7) | 548 (3.1) | 275 (2.7) | 74 (1.7) | 12 (0.4) | 2083 (3.1) |  |
| 2012-2016 | 1425 (4.5) | 1846 (10.3) | 1448 (14.1) | 573 (13.2) | 228 (6.7) | 5520 (8.2) |  |
| 2017-2021 | 3821 (12.1) | 3687 (20.6) | 2978 (29.0) | 1410 (32.6) | 1085 (31.8) | 12981 (19.2) |  |
| 2022-2024 | 25143 (79.6) | 11752 (65.8) | 5549 (54.0) | 2268 (52.4) | 2080 (61.0) | 46792 (69.3) |  |
| **Died** |  |  |  |  |  |  | *** |
| No | 30894 (97.8) | 16725 (93.6) | 9389 (91.4) | 3728 (86.1) | 2839 (83.3) | 63575 (94.2) |  |
| Yes | 700 (2.2) | 1143 (6.4) | 886 (8.6) | 603 (13.9) | 571 (16.7) | 3903 (5.8) |  |
| **HIV Positive** |  |  |  |  |  |  | *** |
| No | 11414 (41.0) | 4837 (36.4) | 2529 (37.6) | 1292 (40.6) | 1415 (47.7) | 21487 (39.8) |  |
| Yes | 0 (0.0) | 4828 (36.3) | 2988 (44.4) | 1524 (47.9) | 1306 (44.1) | 10646 (19.7) |  |
| Not applicable | 15780 (56.7) | 3466 (26.1) | 1068 (15.9) | 264 (8.3) | 173 (5.8) | 20751 (38.4) |  |
| Missing/Unknown | 646 (2.3) | 161 (1.2) | 150 (2.2) | 103 (3.2) | 70 (2.4) | 1130 (2.1) |  |
| **Hypertension** |  |  |  |  |  |  |  |
| No | 31087 (98.4) | 9288 (52.0) | 2042 (19.9) | 367 (8.5) | 106 (3.1) | 42890 (63.6) |  |
| Yes | 0 (0.0) | 8557 (47.9) | 8231 (80.1) | 3963 (91.5) | 3303 (96.9) | 24054 (35.6) | *** |
| Missing | 507 (1.6) | 23 (0.1) | 2 (0.0) | 1 (0.0) | 1 (0.0) | 534 (0.8) |  |
| **Diabetes** |  |  |  |  |  |  |  |
| No | 10232 (32.4) | 9680 (54.3) | 6337 (61.7) | 2344 (54.1) | 1674 (49.1) | 30267 (44.9) |  |
| Yes | 0 (0.0) | 297 (1.7) | 1121 (10.9) | 1332 (30.8) | 1507 (44.2) | 4257 (6.3) | *** |
| Not applicable | 20843 (66.0) | 7814 (43.8) | 2798 (27.3) | 647 (14.9) | 227 (6.7) | 32329 (47.9) |  |
| Missing | 519 (1.6) | 51 (0.3) | 11 (0.1) | 7 (0.2) | 2 (0.1) | 590 (0.9) |  |
| **Stroke** |  |  |  |  |  |  |  |
| No | 4488 (100.0) | 5094 (99.0) | 4069 (95.5) | 2224 (89.8) | 1971 (73.5) | 17846 (93.7) |  |
| Yes | 0 (0.0) | 32 (0.6) | 161 (3.8) | 219 (8.8) | 701 (26.2) | 1113 (5.8) | *** |
| Missing | 2 (0.0) | 17 (0.3) | 31 (0.7) | 34 (1.4) | 8 (0.3) | 92 (0.5) |  |
| **Any Cancer** |  |  |  |  |  |  |  |
| No | 238 (22.8) | 461 (54.7) | 518 (58.1) | 488 (59.2) | 612 (50.2) | 2317 (48.1) |  |
| Yes | 0 (0.0) | 0 (0.0) | 9 (1.0) | 39 (4.7) | 184 (15.1) | 232 (4.8) | *** |
| Not applicable | 296 (28.3) | 341 (40.5) | 338 (37.9) | 271 (32.9) | 388 (31.9) | 1634 (33.9) |  |
| Missing/Unknown | 511 (48.9) | 41 (4.8) | 26 (2.9) | 26 (3.2) | 34 (2.8) | 638 (13.2) |  |
| **Heart Disease/Failure** |  |  |  |  |  |  |  |
| No | 1460 (99.9) | 1963 (99.1) | 1692 (96.1) | 1522 (94.9) | 1997 (91.0) | 8634 (95.9) |  |
| Yes | 0 (0.0) | 9 (0.5) | 37 (2.1) | 48 (3.0) | 178 (8.1) | 272 (3.0) | *** |
| Missing/Unknown | 2 (0.1) | 8 (0.4) | 31 (1.8) | 34 (2.1) | 19 (0.9) | 94 (1) |  |
| **Angina** |  |  |  |  |  |  |  |
| No | 4336 (99.9) | 4921 (98.8) | 3946 (94.0) | 2004 (79.5) | 1208 (41.7) | 16415 (86.7) |  |
| Yes | 0 (0.0) | 40 (0.8) | 223 (5.3) | 484 (19.2) | 1676 (57.9) | 2423 (12.8) | *** |
| Missing/Unknown | 3 (0.1) | 19 (0.4) | 29 (0.7) | 34 (1.3) | 10 (0.3) | 95 (0.5) |  |
| **High Cholesterol** |  |  |  |  |  |  |  |
| No | 4201 (87.4) | 3354 (88.3) | 2117 (73.5) | 1272 (60.1) | 1288 (51.6) | 12232 (76.0) |  |
| Yes | 0 (0.0) | 75 (2.0) | 212 (7.4) | 302 (14.3) | 501 (20.1) | 1090 (6.8) | *** |
| Not applicable | 90 (1.9) | 322 (8.5) | 528 (18.3) | 531 (25.1) | 699 (28.0) | 2170 (13.5) |  |
| Missing | 515 (10.7) | 49 (1.3) | 24 (0.8) | 10 (0.5) | 7 (0.3) | 605 (3.8) |  |
| **Kidney Problem** |  |  |  |  |  |  |  |
| No | 570 (53.0) | 976 (95.2) | 1282 (96.2) | 1314 (92.8) | 1721 (81.9) | 5863 (84.3) |  |
| Yes | 0 (0.0) | 12 (1.2) | 36 (2.7) | 90 (6.4) | 370 (17.6) | 508 (7.3) | *** |
| Missing/Unknown | 506 (47) | 37 (3.6) | 13 (1) | 12 (0.8) | 11 (0.5) | 579 (8.3) |  |
| **TB** |  |  |  |  |  |  |  |
| No | 2289 (8.4) | 2319 (19.1) | 1757 (30.1) | 1326 (48.0) | 1519 (55.2) | 9210 (18.2) |  |
| Yes | 0 (0.0) | 130 (1.1) | 378 (6.5) | 407 (14.7) | 801 (29.1) | 1716 (3.4) | *** |
| Not applicable | 24125 (88.6) | 9333 (77.1) | 3514 (60.1) | 935 (33.8) | 384 (14.0) | 38291 (75.5) |  |
| Missing/Unknown | 807 (3) | 328 (2.7) | 194 (3.3) | 97 (3.5) | 48 (1.7) | 1474 (2.9) |  |
| **Liver Disease** |  |  |  |  |  |  |  |
| No | 151 (99.3) | 192 (97.0) | 167 (84.3) | 106 (76.3) | 57 (74.0) | 673 (88.1) |  |
| Yes | 0 (0.0) | 0 (0.0) | 1 (0.5) | 1 (0.7) | 7 (9.1) | 9 (1.2) | *** |
| Missing | 1 (0.7) | 6 (3.0) | 30 (15.2) | 32 (23.0) | 13 (16.9) | 82 (10.7) |  |
| **Asthma** |  |  |  |  |  |  |  |
| No | 90 (95.7) | 317 (94.9) | 552 (92.9) | 620 (88.6) | 872 (72.5) | 2451 (83.8) |  |
| Yes | 0 (0.0) | 2 (0.6) | 10 (1.7) | 45 (6.4) | 280 (23.3) | 337 (11.5) | *** |
| Refused | 0 (0.0) | 1 (0.3) | 0 (0.0) | 1 (0.1) | 1 (0.1) | 3 (0.1) |  |
| Missing/Unknown | 4 (4.3) | 14 (4.2) | 32 (5.4) | 34 (4.9) | 49 (4.1) | 133 (4.5) |  |
| **Thyroid** |  |  |  |  |  |  |  |
| No | 237 (100.0) | 448 (100.0) | 513 (99.0) | 497 (97.8) | 653 (92.5) | 2348 (97.1) |  |
| Yes | 0 (0.0) | 0 (0.0) | 4 (0.8) | 9 (1.8) | 45 (6.4) | 58 (2.4) | *** |
| Missing/Unknown | 0 (0.0) | 0 (0.0) | 1 (0.2) | 2 (0.4) | 8 (1.2) | 11 (0.5) |  |
| **Obese** |  |  |  |  |  |  |  |
| No | 13665 (100.0) | 8232 (73.0) | 2756 (33.1) | 1050 (27.9) | 717 (23.3) | 26420 (65.9) |  |
| Yes | 0 (0.0) | 3041 (27.0) | 5570 (66.9) | 2719 (72.1) | 2354 (76.7) | 13684 (34.1) | *** |
| **Dyslipidemia** |  |  |  |  |  |  |  |
| No | 4283 (89.1) | 2903 (74.9) | 1349 (42.9) | 446 (17.7) | 137 (4.4) | 9118 (52.1) |  |
| Yes | 0 (0.0) | 912 (23.5) | 1749 (55.7) | 2047 (81.0) | 2979 (95.0) | 7687 (44.0) | *** |
| Not applicable | 8 (0.2) | 24 (0.6) | 33 (1.1) | 27 (1.1) | 19 (0.6) | 111 (0.6) |  |
| Missing | 515 (10.7) | 38 (1.0) | 11 (0.4) | 6 (0.2) | 1 (0.0) | 571 (3.3) |  |
| **Anaemia** |  |  |  |  |  |  | *** |
| No | 329 (100.0) | 469 (98.3) | 622 (95.1) | 691 (91.3) | 917 (77.5) | 3028 (89.1) |  |
| Yes | 0 (0.0) | 8 (1.7) | 32 (4.9) | 66 (8.7) | 266 (22.5) | 372 (10.9) |  |
| *** p<.001. ** p<.01. * p<.05 |  |  |  |  |  |  |  |

Figure S3: The top 25 common multimorbidity patterns across seven chronic conditions.

1. PY – Person Years, HDSS – Health and demographic surveillance system. [↑](#footnote-ref-1)
2. Causes of death derived from verbal autopsy data using InterVA-5 [↑](#footnote-ref-2)
