# Supplementary material for: Data Resource Profile: Multimorbidity in Africa Digital Innovation, Visualisation and Application (MADIVA) research hub
Source: Int J Epidemiol. 2026 Jul 25;55(4):dyag124. doi: 10.1093/ije/dyag124 (PMC13401479; doi:10.1093/ije/dyag124)
Supplement: dyag124_Supplementary_Data [file dyag124_supplementary_data.zip › ije-2025-11-2161-File006.docx]

**Supplementary Table S1: Indicator domains^[[1]](#footnote-1)^ and their availability from harmonised studies**

| **Domain** | **Indicator** | **measurement** | **HAALSI (1,2,3)** | **Assess-1-main** | **SU Population Baseline & Endline** | **HIV/ NCD** | **AWIGEN** | **SU follow up clinic** | **Diabetic** | **Nkateko (1,2)** | **Individual health (1,2)** | **ARK Study (1,2)** |
| --- | --- | --- | --- | --- | --- | --- | --- | --- | --- | --- | --- | --- |
| Demographic | age in years  computed from  (date of interview and date of birth) | <20, 20-29, 30-39, 40-49, 50-59, 60-69, 70+ | x | x | x | x | x | x | x | x | x | x |
|  | sex | male, female | x | x | x | x | x | x | x | - | x | x |
| Body measurements | height | height in cm | x | x | x | x | x | - | x | - | x | x |
|  | weight | weight in kg | x | x | x | x | x | - | x | - | x | x |
|  | waist | waist in cm | x | x | x | x | x | - | x | x | - | x |
|  | hip | hip in cm | x | x | x | x | x | x | x | - | - | x |
| Lifestyle | **Tobacco** | |  |  |  |  |  |  |  |  |  |  |
|  | smoking status | (yes, no) ever, current | x | x | x | x | x | - | x | x | - | - |
|  | smoking frequency | number of cigarettes smoked per days | x |  |  | x | x |  | x | x | - | x |
|  | type of tobacco | pipe, snuff, cigarettes, chewing | x |  | x | x | x |  | x | - | - | x |
|  | age at start | age in years | x | x | x | x | x |  | - | - | - | x |
|  | age at stop | age in years | x |  | x | - | x |  | - | - | - |  |
|  | stopped smoking | years |  |  | x |  |  |  | x |  |  |  |
|  | **Alcohol** | |  |  |  |  |  |  |  |  |  |  |
|  | drinking status | (yes, no) ever, current | x |  | x | x | x |  | x | - | - | - |
|  | alcohol type | spirit, wine, beer, home brew, other | x |  | x | x | x |  | x | - | - | - |
|  | drinking frequency | amount of alcohol in the past 7days, 30 days, 12 months, | x |  | x | x | x |  | - | - | - | - |
|  | number of bottles | less than 1, 2-5+, binge drinking | x |  | x | x | x |  | - | - | - | - |
|  | **Activity (Exercise)** | |  |  |  |  |  |  |  |  |  |  |
|  | moderate sports | minutes, hours, days, | x |  |  | x | x |  | - | - | - | - |
|  | vigorous intensity | minutes, hours, days | x |  | x | x | x |  | - | - | - | - |
|  | sitting | minutes, hours, sitting or standing | x |  |  | x | x |  | - | - | - | - |
|  | sleeping | hours of sleep | x |  |  | - | - |  | - | - | - | - |
|  | vigorous sports | minutes, hours, days | x |  | x | x | x |  | - | - | - | - |
|  | walking/bicycling | minutes, hours, days | x |  | x | x | x |  | x | - | - | - |
|  | **Advice** | |  |  |  |  |  |  |  |  |  |  |
|  | diet change | change diet | x |  | x | x | - |  | x |  | - | - |
|  | exercise | Advice on exercise, Advice to start or do more exercise during the last twelve months | x |  | x | x | - |  | x | - | - | - |
|  | stop smoking | due to diabetic, due to hypertension | x |  | x | x | - |  | - | - | - | - |
|  | weight loss | due to diabetic, due to hypertension |  |  | x | x | - |  | - | - | - | - |
|  | **Diet and Food** | |  |  |  |  |  |  |  |  |  |  |
|  | juice/ soft drinks | number of drinks per week, 12 months | x |  | x | x | x |  | - | - | - | - |
|  | fruit and vegetable consumption | days, number of serving fruits | x |  | x | x | x |  | - | - | - | - |
|  | diet change | reduced salts intake due to hypertension | x |  | x | x | x |  | - | - | - | - |
| Infectious disease | **HIV** | |  |  |  |  |  |  |  |  |  | **-** |
|  | HIV care | counselling status | x |  | - | - | x |  | - | - | - | x |
|  | treatment | (yes, no) current, ever | x |  | - | - | x | - | - | - | x | x |
|  | status | self-reported (yes, no) | x |  | - | - | x | x | - | - | x | x |
|  | test result | negative, positive | x |  | - | - | x | - | - | - | x | x |
|  | history of HIV testing | month, year | x |  | - | - | x | - | - | - | - | x |
|  | **Bilharzia/Schistosomiasis** | |  |  |  |  |  |  |  |  |  |  |
|  | bilharzia status | never, ever | - |  | - | - | - | - | - | - | - | x |
|  | treatment | current, frequency | - |  | - | - | - | - | - | - | - | x |
|  | test | ever tested, urine test results | - |  | - | - | - | - | - | - | - | x |
|  | **Tuberculosis (TB)** | |  |  |  | **-** |  |  |  |  |  |  |
|  | tuberculosis status | last 12 months, | x |  | - | - | x | x | - | - | x | - |
|  | counselling status | (no , yes) | x |  | **-** | **-** | **x** | **-** | **-** | **-** | **x** | **-** |
|  | history | never, ever | x |  | - | - | x | - | - | - | x | - |
|  | treatment | current, ever | x |  | - | - | x | - | - | - | x | - |
|  | **Malaria** | |  |  |  |  |  |  |  |  |  |  |
|  | history | ever, last 12 months | - |  | - | - | x | - | - | - | - | - |
|  | **Sexually Transmitted Diseases** | |  |  |  |  |  |  |  |  |  | **-** |
|  | chancroid | infection status | - |  | - | - | x | - | - | - | - | - |
|  | genital herpes | infection status | - |  | - | - | x | - | - | - | - | - |
|  | pelvic inflammatory disease | infection status | - |  | - | - | x | - | - | - | - | - |
|  | syphilis infection s | infection status | - |  | - | - | x | - | - | - | - | - |
|  | trichomonas infection | infection status | - |  | - | - | x | - | - | - | - | - |
| Sexual Health and Behavior | **Sex** | |  |  |  |  |  |  |  |  |  |  |
|  | transaction sex | ever had sex in exchange for money; was condom used, | x |  | - | - | - | - | - | - | - | - |
|  | sex initiation | age at first sex | x |  | - | - | - | - | - | - | - | - |
|  | choice | chose to start sex | x |  | - | - | - | - | - | - | - | - |
|  | sexual violence | ever forced, forced sex without condom | x |  | - | - | - | - | - | - | - | - |
|  | sexual status | never, ever | x |  | - | - | - | - | - | - | - | - |
|  | sexual partners | total number of sexual partners in lifetime, | x |  | - | - | - | - | - | - | - | - |
|  | reasons to start sex | choice, circumstance | - |  | - | - | - | - | - | - | - | - |
|  | sex with HIV partner | no condom, condom | x |  | - | - | - | - | - | - | - |  |
| Cardiovascular |  |  |  |  |  |  |  |  |  |  |  |  |
|  | **Angina** | |  |  |  |  |  |  |  |  |  |  |
|  | Angina status | ever (no, yes) | x |  | x | x | x | x | - | - | - | x |
|  | family history | (parents, sibling, children) |  |  | - |  |  | - | - |  |  |  |
|  | symptoms | pain or discomfort (lower chest, upper chest, left arm), symptoms of angina past 2 weeks | x |  | x | x | x | - | - | - | - | x |
|  | **Asthma** | |  |  |  |  |  |  |  |  |  |  |
|  | Asthma status | ever, family history (mom, dad) | x |  | - | - | x | - | - | - | - | - |
|  | **Heart Attack** | |  |  |  |  |  |  |  |  |  |  |
|  | Heart attack status | ever (yes,no), | x |  | x | x | x | x | - | x | - | x |
|  | diagnosed | other heart disease, year | x |  | - | - | x | - | x | x | - | - |
|  | treatment | ever received, currently receiving, | x |  | - | - | x | - | - | x | - | x |
|  | traditional treatment | ever received, currently receiving, | x |  | - | - | x | - | - | x | - | x |
|  | family history | mother, father, blood sibling (diagnosed and or treated**)** | x |  | - | - | - | - | - | x | - | - |
|  | **Stroke** | |  |  |  |  |  |  |  |  |  |  |
|  | stroke status | Ever (yes, no) | x |  | x | x | - | x | x | x | - | x |
|  | symptoms and Impact on Life | numbness one side, weakness one side, interfering daily activities, visual impairment | x |  | x | x | - | - | - | - | - | - |
|  | family history | mother, father, blood sibling (diagnosed and or treated**)** |  |  | - |  |  | - | - | x | - | - |
|  | diagnosed | year diagnosed, | x |  | x | x | - | - | - | - | - | - |
|  | onset of paralysis | Greater than 24 hrs., | x |  | x | x | - | - | - | - | - | - |
|  | treatment | medication last 2 weeks, 12 months | x |  | x | x | - | - | - | x | - | x |
|  | **Chest Pain** | |  |  |  |  |  |  |  |  |  |  |
|  | difficult breathing | Ever, current |  |  | - | x | x | x | x | - | - | - |
| Metabolic | **Blood Pressure** | |  |  |  |  |  |  |  |  |  |  |
|  | measurement status | Ever been measured for BP | x |  | x | x | x | x | x | x | x | - |
|  | diastolic | millimeters of mercury (mmHg) | x |  | x | x | x | x | x | x | x | x |
|  | systolic | millimeters of mercury (mmHg) | x |  | x | x | x | x | x | x | x | x |
|  | pulse | pulse (BPM) | x |  | x | x | x | x | - | x | x | x |
|  | treatment | yes, no | x |  |  |  | x | - | - | - | x |  |
|  | **Cholesterol related** | |  |  |  |  |  |  |  |  |  |  |
|  | cholesterol status | ever (yes,no), cholesterol measurement | x |  | - | x | x | - | - | x | - | x |
|  | family history | children, mom, sibling, dad, family | - |  | - | x | - | - | - | - | - | x |
|  | treatment | past 2 weeks, currently, | x |  | - | x | x | - | - | - | - | x |
|  | **Diabetes** | |  |  |  |  |  |  |  |  |  |  |
|  | diagnosed | ever (no,yes), newly diagnosed in the past 12 months, | x |  | x | x | x | x |  | x | x | x |
|  | family history | children, mom, sibling, dad, family |  |  | - |  | x | - | x | - | - | x |
|  | treatment | ever been treated, current | x |  | x | x | x | - | x | x | x | x |
|  | type of treatment | insulin, pills, weight loss | x |  | x | x | x | - | x | x | - | - |
|  | insulin use | today, past 2 weeks, 12 months | x |  | x | x | x | - | x | x | - | - |
|  | traditional  treatment | ever received, currently receiving, |  |  | - | x | x | - | x | x | - | - |
|  | advice and lifestyle adjustment | exercise, weight loss, stop smoking | x |  | x | x | - | - | x | - | - | - |
|  | **Hypertension** | |  |  |  |  |  |  |  |  |  |  |
|  | hypertension status | ever (yes, no), duration | x |  | x | x | x | x | x | x | - | x |
|  | family history | children, dad, mom, parents, sibling | - |  | - | - |  | - | - | - | - | - |
|  | treatment | ever been on treatment, current, 2 weeks, 12 months | x |  | x | x | x | - | x | x | - | x |
|  | tradition  treatment | currently (yes, no) | - |  | - | x | x | - | x | - | - | - |
|  | **Kidney** | |  |  |  |  |  |  |  |  |  |  |
|  | kidney disease status | ever (yes, no) | - |  | x | - | x | x | x | - | - | x |
|  | history and measurement | kidney low function, kidney test ever |  |  | - |  |  | - | – | - | - | x |
|  | kidney disease family history | children, dad, mom, parents, other, parents, sibling | - |  | - | - | x | - | - | - | - | x |
|  | diagnosed | year diagnosed with kidney | - |  | - | - | - | - | - | - | - | x |
|  | treatment | receiving kidney disease medication, | - |  | - | - | - | - | - | - | - | - |
|  | kidney stones | History of kidney stones in family, parents, sibling, mom, other, number of occurrences, | - |  | - | - | x | - | - | - | - | x |
|  | **Obesity** | |  |  |  |  |  |  |  |  |  |  |
|  | family history | history of weight problem/obesity (father, mother) | - |  | - | - | x | - | - | - | - | - |
| Cancer | **Cancer Types** | |  |  |  |  |  |  |  |  |  |  |
|  | breast cancer | family history (mother), ever had breast cancer | x |  | x | - | x | x | - | - | - | - |
|  | cervical cancer | family history (mother), ever had breast cancer | x |  | x | - | x | x | - | - | - | - |
|  | prostate cancer | family history (father), ever had prostate cancer | x |  | x | - | x | x | - | - | - | - |
|  | other cancer types | history of unspecified cancers | x |  | x | - | x | x | - | - | - | x |
|  | treatment | treatment for other cancers | x |  | - | - | x | - | - | - | - | - |
| Other | **Other indictors** | |  |  |  |  |  |  |  |  |  |  |
|  | health service utilization | attended private clinic in the past 3 months, ever attended a public clinic, attended public clinic in the past 3 months | x |  | x | - | x |  | x | x | x | - |
|  | screening for chronic conditions | Tb, cancer, other |  |  | - |  |  |  | x | - | - | x |
|  | medications and treatments | any medication received in the past 12 hours, current medication for chronic condition, ever received medication for chronic condition, | x |  | - | x | x |  | - | x | - | x |
|  | traditional medication | in the past 12 months, ever received traditional medication for chronic condition | - |  | - | x |  |  | - | - | x | x |
|  | personal and lifestyle factors | average sleep duration, self-reported health rating, | x |  | - | - | x |  | - | - | - | - |
|  |  | injury history, |  |  | - | - | x |  | - | - | - | - |
|  |  | history of injected drug use, |  |  | - | - | - |  | - | - | - | - |
|  |  | contraception usage, |  |  | - | - | x |  | - | - | - | x |
|  |  | pesticide exposure, region of pesticide exposure. |  |  | - | - | x |  | - | - | - | - |
|  | reproductive health | | x |  |  | - |  |  |  |  |  |  |
|  | menstrual period | month of last menstrual period, year of last menstrual period, day of menstrual stopping,  current menstrual status, | - |  | - | - | - |  | - | - | - | x |
|  | pregnancy | pregnancy status, number of pregnancies, number of births, number of children. | x |  | - | x | x |  | - | - | - | x |
|  | circumcision | circumcision status, age at circumcision, time of circumcision, place of circumcision | x |  | - | x | - |  | - | - | - | - |
|  | **Other Health Conditions** | |  |  |  |  |  |  |  |  |  |  |
|  | liver disease | history of liver disease | - |  | x | - | x |  | - | - | - | - |
|  | convulsion | convulsion history | x |  | - | - | - |  | - | - | - | - |
|  | seizure | seizure history | x |  | - | - | - |  | - | - | - | - |
|  | vision | vision problems, self-reported poor vision | x |  | - | - | - |  | - | - | - | - |
|  | fever | current fever | x |  | - | - | - |  | - | - | - | - |
|  | **Specimen Information** | |  |  |  |  |  |  |  |  |  |  |
|  | specimen | hour specimen taken, minute specimen taken | - |  | - | x | x |  | - | - | x | x |

1. Additional details with specific variable names are available at https://github.com/MADIVA-DSI/data/blob/main/documentation/madiva_data_specification.xlsx [↑](#footnote-ref-1)
